# The Relationship of Motor Coordination, Visual Perception, and Executive Function to the Development of 4–6-Year-Old Chinese Preschoolers' Visual Motor Integration Skills

**DOI:** 10.1155/2017/6264254

**Published:** 2017-12-31

**Authors:** Ying Fang, Jingmei Wang, Ying Zhang, Jinliang Qin

**Affiliations:** Hangzhou College of Early Childhood Teachers' Education, Zhejiang Normal University, Hangzhou 310012, China

## Abstract

Visual motor integration (VMI) is a vital ability in childhood development, which is associated with the performance of many functional skills. By using the Beery Developmental Test Package and Executive Function Tasks, the present study explored the VMI development and its factors (visual perception, motor coordination, and executive function) among 151 Chinese preschoolers from 4 to 6 years. Results indicated that the VMI skills of children increased quickly at 4 years and peaked at 5 years and decreased at around 5 to 6 years. Motor coordination and cognitive flexibility were related to the VMI development of children from 4 to 6 years. Visual perception was associated with the VMI development at early 4 years and inhibitory control was also associated with it among 4-year-old and the beginning of 5-year-old children. Working memory had no impact on the VMI. In conclusion, the development of VMI skills among children in preschool was not stable but changed dynamically in this study. Meanwhile the factors of the VMI worked in different age range for preschoolers. These findings may give some guidance to researchers or health professionals on improving children's VMI skills in their early childhood.

## 1. Introduction

Visual motor integration (VMI) can be defined as the extent to which visual perception and finger-hand movements are well coordinated [1]. As a vital assessment for child development, many studies found that the VMI skills had a positive influence on fine motor development [2, 3] and self-regulation [4]. Moreover it could predict the further handwriting performance [5–7] and academic achievements [8–10]. Many researchers reported that the VMI skills of western children developed quickly in the early childhood and the trajectory of VMI development among western children was consistent before 3 years, but was inconsistent from 3 to 7 years [1, 11, 12].

However the inconsistence of VMI development during the early childhood was different in China [3, 13, 14]. The trajectory of VMI development among Chinese children was consistent before 4 years but was inconsistent after that. For example, Zhang's team [13] found that the VMI skills of children grew slowly from 4 to 6 years but rapidly developed in the next two years in Beijing, China, by using the Beery Visual Motor Integration 4th Edition (Beery VMI-4), while Cui and his colleagues [14] reported that the fast developing period of VMI was between 4 and 5 years and the slow-down period was between 6 and 7 years using Beery VMI-4 in Shanghai and Ningbo, the east of China. Meanwhile Ng et al.'s studying in Hong Kong depending upon the Beery VMI-5 [3] showed that the VMI increased significantly fast between 4 and 4.5 years and decreased quickly between 4.5 years and 5 years. In general, the VMI skills of children in early childhood developed dynamically.

In addition, there are some researches exploring the factors influencing the VMI development among children in prekindergarten (aged 53–67 months) and kindergarten in the western countries, but few studies on the factors affecting Chinese children's VMI development are found [1, 12, 15]. According to the previous studies, visual perception (VP), motor coordination (MC), and executive function (EF) were probably associated with the VMI development [1, 12, 15].

Firstly, as one of two main parts of the VMI abilities according to Beery VMI-6 [1], the VP was identified as the ability to receive, recognize, analyze, and elaborate the visual stimulus from objects and events [1]. It contained visual recognition and discrimination, visual memory, visual spatial orientating and processing, and perception of figures. Particularly the spatial perception of children in early childhood was considered as the important part of perception in Vereecken's study [15]. In Vereecken's study on children's spatial perception and representation, the visual spatial skills of children before 5 years were at the topology level. After that the skills developed to understand different spatial relationships, for example, approach and separation, dot and plane [15]. Moreover comparing children in United States, Chinese children were immersed in the atmosphere of Chinese characters, which contained complex graphical structures by nature [3, 14]. Thus VP skills would be particularly related to the development of VMI skills in Chinese preschoolers.

Secondly, as another main part of VMI skills, the MC was related to the rebuilding of the representation in mind during the visual motor integration process [1]. Fine motor coordination was proposed to interact with the VMI development which is accompanied with children's fine motor development that began from reaching and grasping to the usage of eating tools, then to the holding of drawing and writing instruments in early years [12, 16, 17]. For Chinese children, the interaction was probably embodied in the usage of life-based tools, for example, chopsticks. They were used by Chinese children from about 4 years according to the guidelines for the learning and development of 3–6-year-old children [18]. It meant that Chinese children had more abundant experience on fine motor earlier. Therefore, the MC skills probably had a great impact on the VMI skills in Chinese preschoolers.

Thirdly, executive function contained three subcomponents that were inhibitory control, working memory, and cognitive flexibility. There were some researches which focused on the relationship between inhibitory control, working memory, and the VMI skills, while there were few on cognitive flexibility [10, 19, 20]. Possibly it was attributed to the fact that cognitive flexibility was not self-existent but to some extent contained inhibitory control and working memory [19]. Moreover higher inhibitory control skills were probably associated with the better VMI [20], although different opinions still existed in the relation between working memory and the VMI skills. For instance, Decker and his team [10] found that the correlation between working memory and the VMI was not significant depending upon verbal or nonverbal ones among children from 4–7 years. However, Becker and his group [20] reported that inhibitory control and working memory were significantly related to the visual motor skills of children in prekindergarten and kindergarten. Consequently, the inconsistence of the relation between working memory and the VMI possibly was attributed to the various tasks. Repeating last word of one sentence task was used in Decker et al.'s study [10] while repeating numbers in order task was used in Becker et al.'s research [20]. The former one would be more difficult for children from 4 to 7 years. If they wanted to have a good performance, they had to learn many words before. Then in the latter one, the order of numbers was really hard for children to remember. The two tasks might have challenged children; thus the present study would choose pictures as stimuli in the working memory task.

Consequently, the present study firstly aimed at exploring the development of the VMI skills, especially the inconsistence of VMI development during early childhood. Moreover, considering the inconsistent age period of the VMI development and the preschool attending law in Mainland China (children in China will usually attend preschools from 3 to 6 years and go to primary schools at 6 and half years, and preschool education is not brought into the compulsory education), children in this study were from 4 to 6 years in preschool. Then the age range was usually one year in the previous studies. But it was a rapid change period in early childhood; thus the present study would add half-year age dividing in the analysis so that the age could be divided more accurately. Furthermore, we firstly hypothesized that the rapid growth period was from 4 to 5 years and the slowing-down period was from 5 to 6 years.

In addition, many researches focused on different factors related to the VMI skills, but visual perception ability, motor coordination, and executive function were studied separately. Moreover there were few studies exploring those factors among Chinese children. Furthermore, the relation of the VMI and cognitive flexibility of EF was less studied and the inconsistence of the correlation between working memory and the VMI existed. Thus the present study would explore the three factors within one study combining the testing of VMI skills in Chinese preschoolers. The second hypothesis was that the visual perception, motor coordination, and executive function all would be associated with the VMI skills among Chinese preschoolers.

## 2. Method

### 2.1. Participants

Children were recruited from 4 preschools in a middle-sized city in Mainland China with their parents' permission and the approvals from the directors of preschools (*N* = 151, *M* = 62.11 months, SD = 8.55, 70 girls, 81 boys). Children were 4 to 6 years old (4 years old: *n* = 60, *M* = 53.20 months, SD = 3.75, 30 girls, 30 boys; 5 years old: *n* = 61, *M* = 65.16 months, SD = 3.38, 25 girls, 36 boys; 6 years old: *n* = 30, *M* = 73.73 months, SD = 1.48, 15 girls, 15 boys). The data was collected between December 2016 and February 2017. 17 additional participants were tested but excluded because of obvious anxiety (*N* = 2 at 4 years old) and half-stopping (*N* = 1 at 4 years old; *N* = 14 at 6 years old). Meanwhile one child did not finish the tasks because he disliked the game, while the other 14 children had not completed the VP, MC, and EF tasks because they had to rehearse their play of graduation ceremony. Tests of the 14 children were planned after their ceremony but they had graduated from preschool at that time. All participants were right-handed and had no history of visual or neurological abnormalities.

### 2.2. Materials

#### 2.2.1. Visual Motor Integration Task (VMI Task)

**Figure 1:**
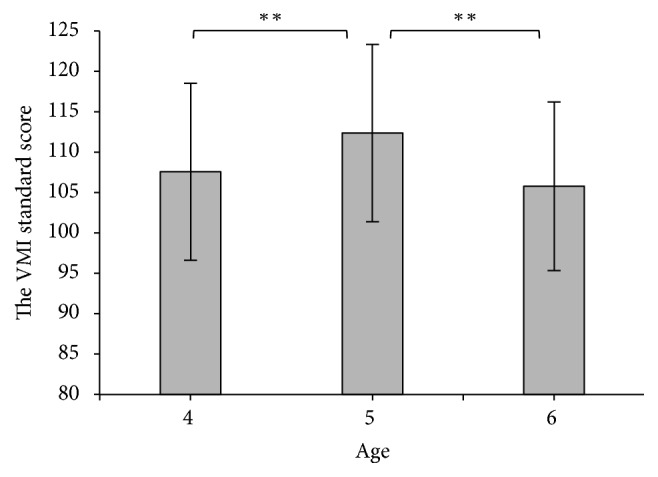
Mean standard score of VMI standard scores of children at three age groups, ^*∗∗*^*p* < 0.01.

The Beery Visual Motor Integration 6th Edition [21] was used to measure the integration of visual perception and motor skills as the participant children imitated and copied a developmentally sequential series of geometric forms using pencil and paper. As the task progressed, the levels of difficulty in geometric forms increased. Totally 30 trials and raw scores were calculated based on the number of correctly recreated figures (1 point), for a possible range between 0 and 30. The standard score was used in analysis according to standard score transforming sheets. The VMI was valid and reliable when the reliability coefficient alpha values ranged between 0.83 and 0.91.

#### 2.2.2. Visual Perception Task (VP Task) and Motor Coordination Task (MC Task)

As one of the supplemental tasks in Beery VMI-6 [1], the VP task was used to measure an individual's visual abilities without the integration of fine motor skills. Children were asked to point to the item in the array, which was identical to the target figure. The choices were written on answer sheets by experimenters or children themselves. Elapsed time was recorded beginning with Item 7, and the task discontinued after 3 minutes. As another supplemental task in Beery VMI-6, the MC task specifically evaluated the fine motor skills rather than its integration with visual perceptual abilities. Children were told to connect dots and to draw within provided borders. Elapsed time was recorded beginning with Item 7 and the task ended up after 5 minutes. The number of valid drawings was calculated. Both tasks had high reliability and validity with test-retest reliability coefficients of 0.84 for VP and 0.85 for MC [1]. In total, each task contained 30 trials and raw scores were calculated based on the number of correctly trials. The possible range was from 0 to 30 and the standard scores were used in analysis according to standard score transforming sheets.

#### 2.2.3. Executive Function Task (EF Task)

Working memory, inhibitory control, and cognitive flexibility were the three subcomponents of EF in this task. Assessments were administrated in a Latin Square design in order to control order effects.

*(1) Working Memory (WM)*. The working memory task was adapted from the classic self-ordered-pointing-task paradigm [22]. In the task, children were required to remember the pictures and their location in a book. First, children were asked to choose one of two different pictures on Page 1. Then, they needed to point out which one has not been chosen when they turned to Page 2 with the same pictures but in a different order. After that, one picture would be added on Page 3 but the order of three pictures would be changed again, and children still needed to find the one they have not chosen before. The number of pictures would increase and the task contained 15 trials ending up with two wrong choices from children. Each black and white picture was 4 × 4 centimeters with an animal (e.g., rabbits, elephants). There were 15 different animals in the task and a possible range was from 0 and 15.

*(2) Inhibitory Control (IC)*. The Day–Night Stroop task [23] was designed to measure inhibitory control. Children were required to inhibit a predominant response by verbally responding to a picture of the sun as “night” and a picture of the moon as “day.” The task was measured through 30 trials. No response or incorrect response was coded as 0. Self-corrected response was coded as 1. Correct response was coded as 2, with the range of scores from 0 to 60. The Day–Night has been shown to be a reliable and valid assessment in kindergarten children [23, 24].

*(3) Cognitive Flexibility (CF)*. The cognitive flexibility task was adapted from the dimensional change card sorting (DCCS) [25]. Cards (4 × 4 centimeters) with different shapes (triangle, circle, and square) and colors (red, yellow, and blue) were prepared (e.g., red circles, yellow square). Children were required to classify the cards depending upon the shapes or colors. At the beginning, children were asked to classify the cards in terms of one dimension (e.g., red cards). Then they were required to sort out the cards in terms of two dimensions with color and shape (e.g., red triangle). The task ended up with two sequential mistakes. The raw score was the number of correct-classified cards.

### 2.3. Procedure

Children participated in the four tasks with the short breaks between the tasks in a quiet room at the preschool. As required in the manual of Beery VMI-6 [1], the three tasks (VMI task, 30 minutes; VP task, 5 minutes; MC task, 5 minutes) were completed in order, while EF task (10 minutes) was randomly finished before the three tasks or after. After finishing all tasks, each child was told that there were some stickers and he/she could choose one sticker as the present.

### 2.4. Data Analysis

SPSS 20.0 was used to obtain descriptive statistics and perform data analyses. To address the first research aim at exploring the VMI development from 4- to 6-year-old children, univariate analysis and pairwise comparison were conducted to verify gender and age effects. To address the second research aim of how the three factors predict the development of children's VMI among different age, bivariate correlations analysis and linear regression were formed considering the visual perception, motor coordination, EF, gender, and age.

## 3. Results

### 3.1. The VMI Developmental Characteristics of 4- to 6-Year-Old Children

Descriptive statistics for all variables in the current analyses are presented in Table 1. Univariate analysis (see Figure 1) showed the significant main effect on gender and age considering the VMI in the current analyses (*F*(1,145) = 12.74, *p* < 0.001, *η*_*p*_^2^ = 0.081; *F*(2,145) = 6.17, *p* < 0.01, *η*_*p*_^2^ = 0.078), but no interaction effect existed between age and gender (*p* > 0.05). The latter pairwise comparison indicated that the levels of VMI skills among girls were significantly higher than those among boys (*p* < 0.05). In terms of different age groups, 5-year-old children were scored significantly higher in the levels of the VMI skills compared with the 4- and 6-year-old ones (*p* < 0.05).

Meanwhile considering the quick change in the early childhood, more detailed ages were divided by each 6-month interval from 4- to 6-year-old children (see Table 2). Univariate analysis showed the significant main effect on gender and age considering the VMI in the current analyses (*F*(1,141) = 12.46, *p* < 0.001, *η*_*p*_^2^ = 0.081; *F*(4, 141) = 5.60, *p* < 0.001, *η*_*p*_^2^ = 0.137), but no interaction effect existed between age and gender (*p* > 0.05). The latter pairwise comparison indicated that the levels of VMI skills among girls were significantly higher than those among boys (*p* < 0.05). In terms of different age groups (see Figure 1 in Supplemental Materials), the early 4-year-old children (from 4 years to 4 years and 5 months) have significantly lower levels of the VMI skills compared to the late 4-year-old children (from 4 years and 6 months to 4 years and 11 months), the early 5-year-old children (from 5 years to 5 years and 5 months), and the late 5-year-old children (from 5 years and 6 months to 5 years and 11 months) (*p* < 0.05), while the late 5-year-old children (from 5 years and 6 months to 5 years and 11 months) had significantly higher VMI scores than the early 6-year-old children (from 6 years to 6 years and 5 months) (*p* < 0.05).

### 3.2. Predictive Variables and Key Components of the VMI in Different Age Group

To address the second research aim of how the three factors can predict the development of children's VMI among different age, bivariate correlations and linear regressions were used.

Firstly bivariate correlations between variables were measured in the analysis (see Table 3). The VMI was significantly correlated to the VP (*r* = 0.21, *p* < 0.05), MC (*r* = 0.39, *p* < 0.01), IC (*r* = 0.18, *p* < 0.05), and CF (*r* = 0.29, *p* < 0.01).

Then linear regressions were conducted to explore the factors predicting the VMI skills depending on three age intervals. The first one was the whole years from 4 to 6 years (see Table 4), the second one was one year (see Tables 5 and 6), and the third one was half year (see Supplemental Materials). Firstly, for the 4- to 6-year-old age interval, Table 4 showed the results of stepwise regression analysis, indicating that MC (*β* = 0.30, *B* = 0.27, *p* < 0.001), CF (*β* = 0.20, *B* = 0.37, *p* < 0.01), and gender (*β* = 0.18, *B* = 3.93, *p* < 0.05) were rated significantly in predicting the development of the VMI skills among 4- to 6-year-old children.

Secondly, for the one-year age interval, according to stepwise regression analysis in Table 5, MC (*β* = 0.45, *B* = 0.40, *p* < 0.001), CF (*β* = 0.29, *B* = 0.47, *p* < 0.01), and IC (*β* = 0.22, *B* = 0.16, *p* < 0.05) created a significant effect on the VMI skills of 4-year-old children. For the VMI of 5-year-old children, there were no significant factors predicting the VMI except gender (see Table 6). By means of the enter regression analysis, the results indicated that MC (*β* = 0.22, *B* = 0.20, *p* = 0.092) and gender (*β* = 0.22, *B* = 4.90, *p* = 0.093) marginally predicted the VMI skills of 5-year-old children (see Table 6). There was no model that can be generated to predict the VMI of 6-year-old children.

In order to explore the factors deeply, the linear regression was used again for the early 4-year-old (4 years to 4 years and 5 months), the late 4-year-old (4 years and 6 months to 4 years and 11 months), the early 5-year-old (5 years to 5 years and 5 months), and the late 5-year-old (5 years and 6 months to 5 years and 11 months) children. It showed that visual perception also predicted the VMI among the early 4-year-old children (*β* = 0.31, *B* = 0.20, *p* < 0.05). The inhibitory control showed its significant effect on the development of VMI (*β* = 0.38, *B* = 0.21, *p* < 0.05) among the early 5-year-old children (see Supplemental Materials).

## 4. Discussion

Utilizing a sample of Chinese preschool children, the present study found out the trajectory of VMI development among 4- to 6-year-old children and used measures of visual perception, motor coordination, and executive function to examine relationships with VMI. Results partly supported the first hypothesis that the rapid growth period was from 4 to 5 years with the significant increase between 4- and 5-year-old groups. But it was not consistent from 5 to 6 years. For the second hypothesis, motor coordination, visual perception, inhibitory control, and cognitive flexibility had an impact on children's development of VMI, but those factors worked in different ages. Motor coordination was the main predictor of the VMI skills of children from 4 to 6 years; visual perception worked at early 4 years. Cognitive flexibility was also the main predictor of the VMI skills among 4- to 6-year-old children, especially among 4-year-old children. Inhibitory control predicted the VMI development of children at early 5 years. In addition, working memory did not predict the development of VMI skills, which was inconsistent with the hypothesis.

### 4.1. The VMI Developmental Characteristics of 4- to 6-Year-Old Chinese Children

Aimed at Chinese children from 4 to 6 years, the VMI skills grew rapidly in the first two years, which was consistent with Zhang et al. [13] and Cui et al. [14]. Firstly, the rapid physiological growth may probably lead to the increase [13]. Brain areas relating to the VMI were developing quickly such as occipital lobe, precentral motor area [26], and posterior parietal cortex [27]. Secondly, the acquisition of some fundamental motor skills could contribute to the rapid development of VMI, such as grasping, drawing, and using chopsticks [14]. Nevertheless, few evidences were directly related to the decrease of VMI skill among children from 5 to 6 years. Thus more studies should be done in the future and it is also one of the future directions to further explore the development of the VMI among children at the period of the transition from kindergarten to primary school. Briefly it indicated that the development of VMI skills in preschool was not stable but changed dynamically in this study.

### 4.2. Predictive Variables and Key Components of the VMI in Different Age Group

In order to explore the factors influencing the development of the VMI skills, the results revealed that the motor coordination, visual perception, inhibitory control, and cognitive flexibility were predictors of the VMI skills on different ages. Firstly, motor coordination and cognitive flexibility were the main predictors of the VMI, especially the former one. The MC worked sufficiently during the VMI process, in which children held the pen, adjusted the gesture and location, rebuilt representation in their mind by using hands, and corrected their actions to perform better [3]. Meanwhile Rosenbloom and Horton [28] reported that children changed writing action by using the dynamic coordination pattern. This pattern changed from body joints to hand joints and finally to fingers. The last stage would be among 4- to 6-year-old children. Secondly, the previous researches rarely focused on the relationship between cognitive flexibility and the VMI because of its complexity containing two subcomponents of EF (working memory and inhibitory control) at the same time. However, children with better cognitive flexibility probably integrated visual and motor information automatically and could reduce the occupation of cognitive resources so as to deal with other more complex information [20].

Thirdly, visual perception was related to the VMI of children at the early 4 years. Perhaps it might be that the visual spatial ability began to develop at that time. As the part of visual spatial abilities, the skills of distinguishing figures developed from matching and pointing out to naming, and children could recognize shapes at this age [29, 30]. In addition, the linear regression showed that inhibitory control was also associated with the VMI of children at the 4 years and at the early 5 years. Probably it was because children could meet more handwriting stimulus at the beginning of 5 years, such as grasping, drawing, writing own names, and others [31]. If the stimuli were overloaded, they had to inhibit interference in order to pay attention to the targets during the VMI process [13]. However working memory did not affect the VMI in this study, possibly because copying geometric figures in the VMI tasks was not so hard for children to remember; at the same time children could check the geometric figures again if they forgot them. Also it could be attributed to the different tasks. Becker et al. [20] chose numbers to test working memory, but in this study, utilized pictures were much easier for preschoolers to finish.

### 4.3. The Characteristics of the VMI Skills among Chinese Participants

Trying to understand more about the need of higher VMI skills among Chinese children in nature, more evidences were mentioned. Exactly in this study, Chinese children had better VMI skills than those in United States in this study. The difference could be demonstrated through the statistical results considering the Chinese standard score of different ages (see Table 1) and those in the US (scores: 100) [1, 32]. As many researchers indicated the culture difference could probably be the main reasons, for example, the game-like and life-based tools usage (e.g., using chopsticks), parents' education concept [32], and the environment of the unique Chinese words [3, 14], meanwhile these cultural factors might be double sided, which could be challenges as well as chances at the same time.

Firstly, the developmental challenges come from the fine motor in children' plays and daily life. These could be revealed in the game-like and life-based tools usage [3], for example, using chopsticks to eating and transfer beads, beans, and rice, the simplified embroidery game. Using chopsticks is one kind of big challenges for preschoolers. In China, children would be encouraged to use chopsticks from about 5 years in preschool according to guidelines for the learning and development of 3- to 6-year-olds [18]. Moreover some interesting games are designed to support children' attempts to use chopsticks in daily life, such as using them to transfer beads, beans, and rice. At the same time, more challenging games aimed at improving fine motor abilities are popular in preschool of the southern China, such as the simplified embroidery game. Because the south of China is famous for the silk and embroidery in history, teachers there usually design the specific activities for children to simply learn the embroidery. Generally, these also provide lots of chances for children to practice the abilities of VMI in their daily life.

Secondly, under the fine motor cultural background (reading and writing the unique Chinese characters), the developmental expectations of visual motor integration were always high. These could be evident in the atmosphere of the unique Chinese characters. As has been shown in the mental geometry theory of the handwriting of Chinese characters, the dynamic integration happens not only between vision and motor, but also between Chinese words and the writers' whole bodies [33]. Ting and his team [34] supported that Chinese people have unique brain areas relating to the motor area. So Chinese words may contain the motor information, which are projected on the actions of writers. Children in china are immersed in these characters everyday so that they unconsciously have absorbed abundant motor information. However, there are still some challenges existing in children's preparations for writing Chinese words when going to attend to primary schools. On the one hand, Chinese words contain distinct brush strokes, radicals, and structures; those challenge the ability to discriminate between the large numbers of memorized visual forms in reading and writing Chinese [3, 14]. For instance, the number of the words (500 words) in the first Chinese textbook for grade one is counted, and 82.4% of words are more than eight strokes, which would be difficult for children to remember. On the other hand, there are many parents sending children to different courses to gain mastery in printing or writing skill and reinforce knowledge acquisition during later preschool period [28, 32], because parents in China held the concept that their children should not lose at the beginning [32]. A large number of parents push their children to work hard frequently. Consequently, probably the culture difference created the unique environment for Chinese children to develop their VMI skills.

## 5. Limitations and Future Directions

In this paper, there are still some limitations. Firstly, the sample size for 6-year-old group is only half as that for the other 2 age groups. Also the late 6-year-old children did not attend to the study because they should go to primary school at that age according to the compulsory education policy. Sampling errors, instead of the developmental trajectory, possibly could cause the age-based difference between the 6-year-old group and the other 2 groups. In addition, children at 6 years would face the great challenges of school readiness on Chinese handwriting. Thus the development of the VMI among children in primary school would be explored especially those during the transition from kindergarten to primary school period. Secondly, the present study just explored some factors related to the VMI development and other factors would be studied later, for instance, the culture difference, the classroom experience, and teacher-child interaction [35].

## 6. Conclusions and Implications

This study explored the details of the VMI development as well as the predictors of the VMI in different age groups for preschoolers. In conclusion, the development of VMI skills in preschool was not stable but changed dynamically in the study. The factors of the VMI worked in different age range for preschoolers. These findings may give some guidance to researchers or health professionals on improving children's VMI skills in the early childhood. Furthermore, the findings showed some implications for researchers or health professionals. Firstly, the development trajectory and predictors of the VMI should be considered reasonably to avoid aimless and unfit instructions on. Secondly, the development of the VMI in early years is very sensitive and dynamical; also it is associated with different factors at different ages, so researchers, health professionals, parents, and teachers should pay more attention to the developmental challenges of the VMI and find the core factors which are related to the development.

## Figures and Tables

**Table 1:** Descriptive statistics of age, VMI, VP, MC, WM, IC, and CF on one-year interval age.

	Age	VMI	VP	MC	WM	IC	CF
4 years							
*n*	60	60	60	60	60	60	60
Mean	53.20	107.58	111.53	103.82	10.07	44.10	7.33
SD	3.75	10.98	16.10	12.26	4.45	15.20	6.86
Range	48–59	83–141	67–144	80–146	2–15	0–60	0–29
5 years							
*n*	61	61	61	61	61	61	61
Mean	65.16	112.38	117.70	109.61	11.44	50.77	12.00
SD	3.38	10.99	11.45	12.59	4.27	13.93	9.91
Range	60–71	95–138	92–146	71–155	2–15	0–60	0–46
6 years							
*n*	30	30	30	30	30	30	30
Mean	73.73	105.80	115.07	108.37	13.10	54.13	10.53
SD	1.48	10.46	8.94	12.93	3.27	8.84	6.33
Range	72–76	90–138	92–146	71–155	2–15	0–60	0–46

*Note*. Age: in months. VMI: visual motor integration; VP: visual perception; MC: motor coordination; WM: working memory; IC: inhibitory control; CF: cognitive flexibility.

**Table 2:** Descriptive statistics of the VMI skills on half-year interval age.

	*n*	Mean	SD	Range
The early 4 years old	30	103.50	10.39	83–141
The late 4 years old	30	111.67	10.14	86–131
The early 5 years old	30	109.97	7.87	96–123
The late 5 years old	31	114.71	13.05	95–138
The early 6 years old	30	105.80	10.46	90–135

*Note*. VMI: visual motor integration; Range: in months; the early 4 years old: 4 years to 4 years and 5 months old; the late 4 years old: 4 years and 6 months to 4 years and 11 months old; the early 5 years old: 5 years to 5 years and 5 months old; the late 5 years old: 5 years and 6 months to 5 years and 11 months old; the early 6 years old: 6 years to 6 years and 6 months old.

**Table 3:** Bivariate correlations between variables.

Variable	Correlations						
	1	2	3	4	5	6	7
(1) Age	-	-	-	-	-	-	-
(2) Gender	−0.02	-	-	-	-	-	-
(3) VMI	−0.01	0.28^*∗∗*^	-	-	-	-	-
(4) VP	0.14	0.06	0.21^*∗*^	-	-	-	-
(5) MC	0.16^*∗*^	0.25^*∗∗*^	0.39^*∗∗*^	0.24^*∗∗*^	-	-	-
(6) WM	0.26^*∗∗*^	0.03	0.13	0.24^*∗∗*^	0.11	-	-
(7) IC	0.28^*∗∗*^	0.01	0.18^*∗*^	0.25^*∗∗*^	0.22^*∗∗*^	0.17^*∗*^	-
(8) CF	0.18^*∗*^	0.12	0.29^*∗∗*^	0.22^*∗∗*^	0.21^*∗∗*^	0.12	0.13

*Note*. Age: in years. VMI: visual motor integration; VP: visual perception; MC: motor coordination; WM: working memory; IC: inhibitory control; CF: cognitive flexibility; ^*∗*^*p* < 0.05; ^*∗∗*^*p* < 0.01.

**Table 4:** Motor coordination, cognitive flexibility tasks, and gender predicting the VMI skills of 4–6-year-old children.

Variable	*B*	SE	*β*
MC	0.27^*∗∗∗*^	0.07	0.30
CF	0.37^*∗∗*^	0.10	0.20
Gender	3.93^*∗*^	1.68	0.18

*Note*. MC: motor coordination; CF: cognitive flexibility; ^*∗*^*p* < 0.05; ^*∗∗*^*p* < 0.01; ^*∗∗∗*^*p* < 0.001.

**Table 5:** Motor coordination, cognitive flexibility, and inhibitory control tasks predicting the VMI skills of 4-year-old children.

Variable	*B*	SE	*β*
MC	0.40^*∗∗∗*^	0.10	0.45
CF	0.47^*∗∗*^	0.17	0.29
IC	0.16^*∗*^	0.07	0.22

*Note*. MC: motor coordination; CF: cognitive flexibility; IC: inhibitory control; ^*∗*^*p* < 0.05; ^*∗∗*^*p* < 0.01; ^*∗∗∗*^*p* < 0.001.

**Table 6:** Gender and motor coordination tasks were marginally associated with the VMI skills of 5-year-old children.

Variable	*B*	SE	*β*
Gender	4.90^†^	2.87	0.22
VP	−0.09	0.12	−0.09
MC	0.20^†^	0.11	0.22
WM	0.05	0.33	0.20
IC	−0.00	0.10	−0.00
CF	0.23	0.14	0.21

*Note*. VP: visual perception; MC: motor coordination; WM: working memory; IC: inhibitory control; CF: cognitive flexibility; ^†^*p* < 0.1.

## Supplementary Materials

Supplementary Materials Table 1: motor coordination, visual perception predicting visual motor integration skills of the younger 4-year-old children. Table 2: motor coordination predicting visual motor integration skills of the elder 4-year-old children. Table 3: inhibitory control predicting visual motor integration skills of the younger 5-year-old children. Table 4: motor coordination predicting visual motor integration skills of the elder 5-year-old children. Figure 1: mean standard score of VMI standard scores of children at five age groups.
