# Supplementary material for: The Relationship of Motor Coordination, Visual Perception, and Executive Function to the Development of 4–6-Year-Old Chinese Preschoolers' Visual Motor Integration Skills
Source: Biomed Res Int. 2017 Dec 31;2017:6264254. doi: 10.1155/2017/6264254 (PMC5804408; doi:10.1155/2017/6264254)
Supplement: Supplementary Materials — Table 1: motor coordination, visual perception predicting visual motor integration skills of the younger 4-year-old children. Table 2: motor coordination predicting visual motor integration skills of the elder 4-year-old children. Table 3: inhibitory control predicting visual motor integration skills of the younger 5-year-old children. Table 4: motor coordination predicting visual motor integration skills of the elder 5-year-old children. Figure 1: mean standard score of VMI standard scores of children at five age groups. [file 6264254.f1.pdf]

Supplemental materials
of "The Contribution of Motor Coordination, Visual Perception and Executive Function to the Development of 4-6 years old Chinese Preschoolers' Visual Motor Integration Skills"

Ying Fang, [1] **Jingmei Wang,** [1] **Ying Zhang** [1]**, Jinliang Qin** [1]

[1] *Hangzhou College of Early Childhood Teachers' Education, Zhejiang Normal University, 310012, Hangzhou, China*

1.The linear regression was used for the younger 4 years old (4 years to 4 years and 5 months), the elder 4 years old (4 years and 6 months to 4 years and 11 months), the younger 5 years old (5 years to 5 years and 5 months) and the elder 5 years old (5 years and 6 months to 5 years and 11 months). Tables are shown below.

TABLE 1：Motor coordination, visual perception predicting visual motor integration skills of the younger 4 years old children

Variable	*B*	SE	*β*
MC	0.54^{**}	0.13	0.61
VP	0.20^{*}	0.09	0.31

Note: MC=Motor Coordination; VP=Visual perception; the younger 4 years old = from 4 to 4 and 5months years old
$^{*}p<0.05$; $^{**}p<0.01$

TABLE 2：Motor coordination predicting visual motor integration skills of the elder 4 years old children

Variable	*B*	SE	*β*
MC	0.45^{**}	0.13	0.56

Note: MC=Motor Coordination; the elder 4 years old = from 4 and 5months to 4 and 11 months years old
$^{**}p<0.01$

TABLE 3：Inhibitory control predicting visual motor integration skills of the younger 5 years old children

Variable	*B*	SE	*β*
IC	0.21^{*}	0.10	0.38

Note: IC= inhibitory control; the younger 5 years old = from 5 to 5 and 5months years old
$^{*}p<0.05$

TABLE 4: Motor coordination predicting visual motor integration skills of the elder 5 years old children

Variable	*B*	SE	*β*
MC	0.42*	0.17	0.42

Note: MC=Motor Coordination; the elder 5 years old = from 5 and 5months to 5 and 11 months years old

*p<0.05

2. Figure 1: Mean standard score of VMI standard scores of children at five age groups

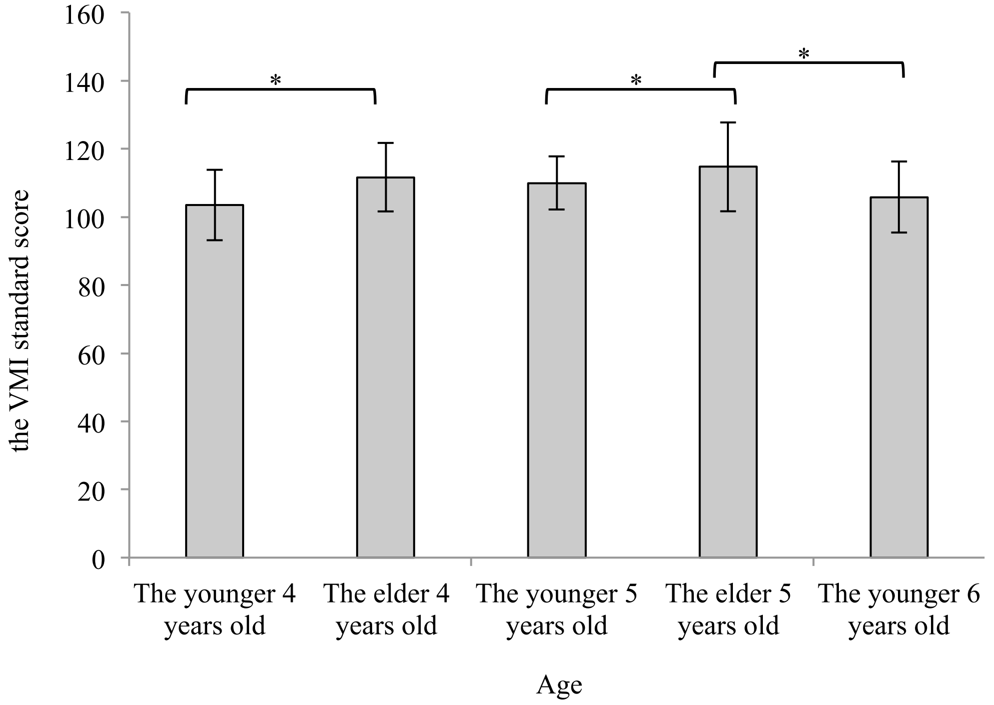

FIGURE1: Mean standard score of VMI standard scores of children at five age groups
*p<0.05
